# Trachoma Prevalence in Four Localities of Darfur Region, Sudan, following One Round of Antibiotic Mass Drug Administration

**DOI:** 10.1080/09286586.2021.1953538

**Published:** 2021-08-21

**Authors:** Balgesa E Elshafie, Mazin Salih Abdalla Elsanosi, Atif El Amin, Robert Butcher, Rebecca Willis, Ana Bakhtiari, Cristina Jimenez, Michael Dejene, Anthony W Solomon, Emma M Harding-Esch, Kamal H Binnawi

**Affiliations:** a Sudan National Trachoma Control Programme, Khartoum, Sudan; b The Carter Center, Khartoum, Sudan; c Clinical Research Department, London School of Hygiene & Tropical Medicine, London, UK; d Taskforce for Global Health, Atlanta, Georgia, USA; e Sightsavers, Haywards Heath, UK; f Sightsavers, Addis Ababa, Ethiopia; g Department of Control of Neglected Tropical Diseases, World Health Organization, Geneva, Switzerland

**Keywords:** Trachoma, trichiasis, Sudan, neglected tropical diseases, elimination, Darfur

## Abstract

**Introduction:**

The prevalence of trachomatous inflammation—follicular (TF) in 1–9-year-olds and of trachomatous trichiasis (TT) in ≥15-year-olds in four endemic evaluation units (EUs) of Darfur region, Sudan, was measured more than a year after the required single round of antibiotic mass drug administration (MDA).

**Methods:**

Surveys were conducted using highly standardised, World Health Organization-recommended methodologies. Individuals aged ≥1 year, resident in selected households, were chosen for the survey using a two-stage cluster sampling process. Consenting adults and children were examined for the signs TF and TT by graders trained to international standards. Prevalence of disease in key indicator groups was calculated and weighted to the underlying population structure.

**Results:**

A mean of 1,415 (range: 1,253–1,611) children aged 1–9 years were examined in each EU. The age-adjusted prevalence of TF in 1–9-year-olds in each of the four surveyed EUs was <5%. A mean of 1,139 people aged ≥15 years (range: 1,080–1,201) were examined in each EU. The estimated age- and gender-adjusted prevalence of TT in ≥15-year-olds was <0.2% in all four EUs. In general, the proportion of households with access to improved WASH facilities was generally lower in this study than in corresponding baseline studies.

**Conclusions:**

No further MDA should be conducted in these four EUs for the next 2 years, at which point they should be re-surveyed to determine whether the prevalence of TF in 1–9-year-olds has remained <5%. Active TT case-finding is also not indicated. Environmental improvement and promotion of facial cleanliness measures should continue to be implemented to prevent disease recrudescence.

## Introduction

Trachoma is the world’s leading infectious cause of blindness.^1^ It is caused by an ocular *Chlamydia trachomatis* infection, presenting initially as a chronic inflammation of the eyelid (which may be sufficiently florid to qualify, in the World Health Organization (WHO) simplified grading system, as trachomatous inflammation—follicular [TF] and/or trachomatous inflammation—intense [TI]^2^). Recurrent infections and inflammation^3^ can lead to trachomatous trichiasis (TT), an in-turning of the eyelashes that brings them into direct contact with the eyeball. TT causes intense pain and can lead to scarring of the cornea and, ultimately, blindness. Visual impairment from trachoma dramatically reduces an individual’s ability to make essential contributions to the family, such as supporting food production, collecting water or caring for children. Furthermore, those blinded from trachoma require support from other members of the household, so the role of the carer in the household is also impacted. Trachoma is strongly associated with and propagates poverty,^4^ with grave medical, economic and social ramifications.

The Sudan National Trachoma Control Programme is making great progress towards the elimination of trachoma as a public health problem. The national plan was updated by the Trachoma Task Force in 2005 to adopt the WHO-recommended SAFE (surgery, antibiotics, facial cleanliness, and environmental improvement) strategy. The initial priority of the programme was to map trachoma in all states of Sudan by conducting population-based trachoma prevalence surveys during 2006–2015 to facilitate planning. The deployed surveys were designed to estimate two key indicators of trachoma burden, namely TF prevalence among children aged 1–9 years and TT prevalence among adults aged ≥15 years, in accordance with WHO indicators for validating trachoma as a public health problem.^5^ From this mapping, approximately 17% of Sudan’s population were identified to be living in areas endemic to trachoma. The prevalence of TF in children aged 1–9 years was as high as 17% and TT in adults aged ≥15 years as high as 3.8%,^6,7^ well in excess of the thresholds at which implementation of the SAFE strategy is recommended.

Sudan continues to face challenges in managing trachoma. For example, some endemic areas are insecure, or inaccessible during the rainy season; these issues hinder service provision. Despite this, in several districts, annual mass drug administration (MDA) of azithromycin, an effective antibiotic^8^ donated by Pfizer through the International Trachoma Initiative for trachoma elimination purposes, has been delivered in line with international recommendations. (Individuals in treated districts who did not qualify for azithromycin were instead offered treatment with topical tetracycline eye ointment.) This involves one MDA round where the TF prevalence in 1–9-year-olds is 5.0–9.9%, and three annual rounds where the TF prevalence is 10.0–29.9%.^9^ At the national level, the programme conducted 91 TT surgical camps, funded by The Carter Center and Sightsavers, between 2011 and 2018. Additional TT surgeries have also been carried out at state hospitals and alongside cataract surgical outreach campaigns. Trachoma health education programmes have also been implemented at community and school levels in targeted localities nationwide (Darfur was not among the targeted localities for these activities). This community health education programme identifies community leaders, teachers, and midwives as focal persons who are subsequently trained in community participation for trachoma elimination work at the community level. The focal person then hosts regular health education sessions and group discussions about trachoma and the SAFE strategy. The focal person will also encourage community members to conduct environmental cleaning campaigns at villages and schools. School health education programmes have also been expanded to include trachoma curricula into basic and secondary schools in all targeted localities. At least one teacher is trained per school to carry out trachoma health education in their school. Trachoma Friendship Societies have been developed to promote health education activities at both the community and school levels, though Darfur region was not included in these activities due to security challenges. It is nevertheless expected that, with proper planning, financial support, and dedication, Sudan can still reach the goal of eliminating trachoma as a public health problem.

The aim of the survey series presented in this manuscript was to measure the prevalence of TF and TT in four localities of the Darfur region, namely Dar El Salam, Kalmindo, El Salam, and Belail. In these localities, the prevalence of TT was 0.0–0.6% and of TF was 5.0–9.9% during baseline mapping in 2014–5^6^ and they duly received a single round of MDA between February and May 2017. Darfur-focussed S, F, and E interventions have not yet begun. The Trachoma Impact Survey (TIS) data generated in this exercise will enable the Sudan National Trachoma Programme to review progress towards elimination in these localities and determine whether further interventions may be needed there in the future.

## Methods

### Study ethics

The study was approved by the Health Research Council in Sudan and the National Research Ethics Review Committee of the Sudan Federal Ministry of Health. The London School of Hygiene & Tropical Medicine Ethics Committee provided approval for Tropical Data to support trachoma surveys (16105).

Communities were contacted in advance of the team’s arrival. Eligible residents were given information about the study in the local language prior to enrolment. Those aged ≥15 years consented verbally for themselves; a parent or guardian consented verbally on behalf of those aged <15 years. Consent for examination was recorded in the Tropical Data app used for data collection. Identified active (inflammatory) trachoma was treated with tetracycline eye ointment, and individuals with significant ocular pathology, including TT, were referred to local services for management.

### Study design

For the purposes of this survey, one evaluation unit (EU) consisted of one locality as that was the administrative unit that most closely met the WHO definition of a district for trachoma elimination purposes (a unit of health administration with a population of 100,000–250,000).^10^ All four EUs (population range: 123,725–267,593) were in the Darfur region of Sudan. Assuming a design effect of 2.63 and applying an inflation factor of 1.2 to account for non-response, a sample of households that included 1164 children aged 1–9 years was required to have 95% confidence of estimating a prevalence of TF of 4% in 1–9-year-olds in each EU with an absolute precision of ±2%.^11^

A two-stage cluster sampling strategy was used for each survey, in-line with previous surveys in Darfur and WHO recommendations.^6,11,12^ The primary sampling unit was defined as a village. Villages were selected from a full list of villages in each EU using a probability-proportional-to-size method. Large villages were subdivided into two or three sectors, which could each be independently selected. The secondary sampling unit was the household. The number of households to be surveyed per village was fixed at 30, based on the number of households a team could feasibly visit in a day. Households were selected through compact segment sampling; subsequent references to “clusters” in this paper refer to those compact segments. All residents aged ≥1 year from selected households were eligible for inclusion in the survey. According to records from the Darfur regional Expanded Programme on Immunization, a mean of 1.6 children aged 1–9 years was expected in each household. The target sample size would therefore be reached after surveying 25 clusters. The precision of the TT prevalence estimate was accepted as that generated by the sample of adults living in the households selected to meet the TF prevalence sample size; we acknowledge that, using a classical frequentist statistical approach, this is imperfect for determining with confidence whether the elimination prevalence target for TT has been met.

### Examination

Grading was undertaken by health-care professionals who had successfully completed the Tropical Data grader training scheme before taking part in the surveys. This included completion of a classroom-based taught course on trachoma, achievement of a kappa score of ≥0.7 in a classroom-based intergrader agreement (IGA) test of 50 photographs, which had previously been graded by international experts, and achievement of a kappa score of ≥0.7 against a Tropical Data-certified trachoma grader trainer in the assessment of 50 eyes of children of whom at least five had TF.^13^ Participants were examined for clinical signs of trachoma using 2.5× binocular loupes and torchlight where necessary. Participants were graded for TF, TI and TT using the WHO simplified grading system with follicle size guides to support diagnosis of TF.^2,14^ In this survey, TT was graded when people were found to have one or more eyelashes touching the eyeball or evidence of recent epilation of in-turned eyelashes as per the original version of the WHO simplified grading scheme published in 1987.^2^ Upper- and lower-eyelid trichiasis were not differentiated. Where a participant was identified with TT, they were additionally assessed for trachomatous scarring (TS), and asked questions about whether they had previously been offered management for their TT and whether they had accepted surgery or epilation.

Data were recorded by dedicated data recorders using the Secure Data Kit-based Tropical Data app (https://www.tropicaldata.org). Data recorders were required to complete a training course on how to recognise different water, sanitation, and hygiene (WASH) service types and how to use the data collection app and then successfully pass an examination of their data entry fidelity. A full suite of quality assurance and quality control measures was implemented.^15^

### Assessment of household water, sanitation, and hygiene infrastructure

Household heads nominated an individual to be asked questions adapted from the United Nations Childrens' Fund (UNICEF)/WHO Joint Monitoring Programme (JMP) for Water Supply and Sanitation WASH household questionnaire.^12,13^ The questionnaire assessed the proximity and status of water and sanitation facilities for each household. The questionnaire also assessed whether there was a handwash station within 15 m of the household latrine. (Handwash station questions were asked where there was an improved or unimproved latrine but not asked when there was no latrine.) Water source type, latrine type, and whether soap and water were available at the handwash station at the time of the visit were assessed by direct observation. Latrine type was categorised as either improved or unimproved, where improved refers to covered latrines in line with UNICEF/WHO JMP definitions.^16^

### Data analysis

Disease prevalence was first calculated at cluster level and weighted according to the underlying population structure. Cluster-level TF prevalence was weighted in 1-year age groups. Cluster-level TT prevalence was weighted in five-year age and gender groups. EU-level disease prevalence was defined as the mean age- (TF) or age- and gender-weighted (TT) cluster-level prevalence from each EU.

To assess whether there was a difference in the likelihood of having TF for those sampled after MDA compared to those sampled before MDA, individual-level data were extracted from previous surveys in these EUs.^6^ A mixed-effects regression model was run with TF as the dependent variable, and whether the individual was sampled before/after MDA, their age and their gender included as fixed-effect independent variables. Cluster of residence was included as a random-effect variable. For the purposes of comparison of pre-MDA data to post-MDA data, data from post-MDA EUs were amalgamated to match pre-MDA EU boundaries. Separate models were run for the North Darfur EUs and the South Darfur EUs.

Mixed-effect regression was also used to determine whether there was any association between TF and individual- and household-level variables. Cluster and household were evaluated as random-effect variables for all models (EU was not evaluated as a random effect variable as there were insufficient levels). Models with household of residence as a random-effects variable failed to converge suggesting overfitting. Therefore, cluster of residence was the only random-effect variable included in all models. The relative contribution of independent variables to overall model fit was calculated by likelihood ratio tests between models with and without the variable of interest. Variables with strong evidence of an association in univariable analysis (*p* < 0.05) were included in the multivariable model. The remaining variables were added to the multivariable model in a stepwise manner to determine whether they improved the model fit and included if they did.

Analyses of the association between TT, age, and gender were not carried out due to the small number of TT cases identified.

## Results

### Study population

Data collection took place in December 2018 and January 2019. Table 1 summarises the number of people recorded as living in selected households and the number who did not take part, separated by reason for non-participation. A total of 12,080 people aged ≥1 year were enumerated across all EUs, of whom 11,248 (93%) were examined. The principal reason for non-participation was being absent at the time of the teams’ visit.

**Table 1.** Proportion of the enumerated population who were enumerated and examined in four evaluation units (EUs) of Darfur region, Sudan, December 2018–January 2019.

State	Locality	EU ID	Pre-MDA prevalence^6^		Age group (years)	Enumerated	Absent	Refused	Other	Examined (response rate, %)	Examined female (%)
			TF in 1–9 years (%)	TT in ≥15 years (%)							
North Darfur	Dar Elsalam	80929	9.3	0.6	1–9	1,266	13	0	0	1,253 (99)	620 (49)
					≥15	1,351	174	1	0	1,176 (87)	796 (68)
					*Total (≥1)*	*2,817*	*188*	*1*	*0*	*2,628 (93)*	*1,526 (58)*
	Kalamindo	80930			1–9	1,357	21	1	1	1,334 (98)	695 (52)
					≥15	1,268	166	1	1	1,100 (87)	765 (70)
					*Total (≥1)*	*2,872*	*190*	*2*	*2*	*2,678 (93)*	*1,603 (60)*
South Darfur	Elsalam	80931	5.2	0.0	1–9	1,506	45	0	0	1,461 (97)	777 (53)
					≥15	1,293	205	7	1	1,080 (84)	779 (72)
					*Total (≥1)*	*3,051*	*276*	*8*	*1*	*2,766 (91)*	*1,675 (61)*
	Belail	80932			1–9	1,629	18	0	0	1,611 (99)	799 (50)
					≥15	1,341	137	3	0	1,201 (90)	902 (75)
					*Total (≥1)*	*3,340*	*161*	*3*	*0*	*3,176 (95)*	*1,927 (61)*

MDA: mass drug administration; TF: trachomatous inflammation – follicular; TT: trachomatous trichiasis.

### Prevalence of disease

**Figure 1.**
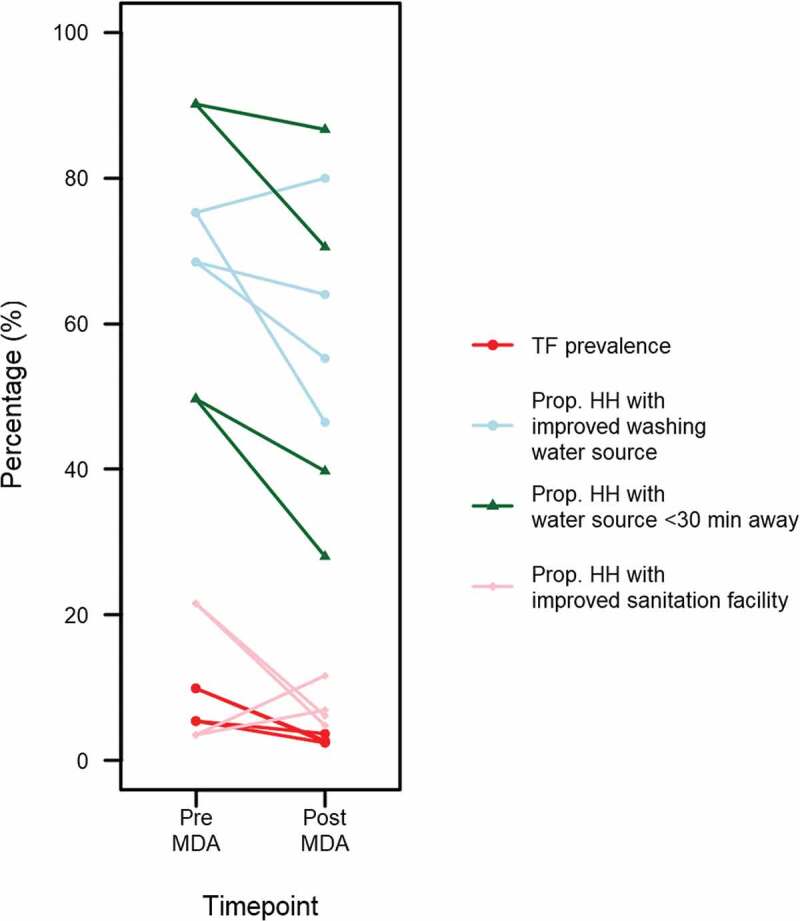
Difference in adjusted prevalence of trachomatous inflammation—follicular (TF) in 1–9-year-olds, proportion of households with an improved washing water source, proportion of households with a washing water source within a 30-minute return journey of the household and proportion of households with an improved latrine in four evaluation units of Sudan. Pre-antibiotic mass drug administration (MDA) survey data collected as part of the Global Trachoma Mapping Project, 2014 − 2015.^6^ Post-MDA data collected in December 2018–January 2019. There are two post-MDA estimates associated with each pre-MDA estimate due to the splitting of each pre-MDA evaluation unit (EU) into two EUs for post-MDA surveys. Prop. HH: Proportion of households

Of 5,659 children aged 1–9 years examined, 197 cases of TF were identified. Only one case of TI was identified in 1–9-year-olds across all four EUs. 125/197 (63%) cases of TF were bilateral. The adjusted prevalence of TF in 1–9-year-olds was <5% in each EU studied (range: 2.5–3.7%; Table 1). The post-MDA TF prevalence was lower than the pre-MDA TF prevalence (Figure 1) and individual-level analyses suggested lower odds of having TF for children sampled after MDA compared to those sampled before MDA (North Darfur EUs adjusted Odds Ratio [aOR]: 0.28, 95% confidence interval [CI]: 0.12–0.60, *p* = 0.002; South Darfur EUs aOR: 0.67, 95% CI: 0.44–1.02, *p* = 0.063). Compared to children aged 1–3 years, children aged 4–6 years and 7–9 years had lower odds of having TF (proportion of children with TF per age bracket in years: 1–3: 5%, 4–6: 3.5%, 7–9: 1.8%; aORs: 1–3 years versus 4–6 years: 0.62, 95% CI: 0.44–0.86; 1–3 years versus 7–9 years: 0.31, 95% CI: 0.20–0.47; *p* = 0.007; Supplementary Table 1). Children living in a household with ≥4 children aged 1–9 years had higher odds of having TF than children living in households with 1–3 children (aOR: 1.52, 95% CI: 1.12–2.06; *p* < .001; Supplementary Table 1).

**Figure 2.**
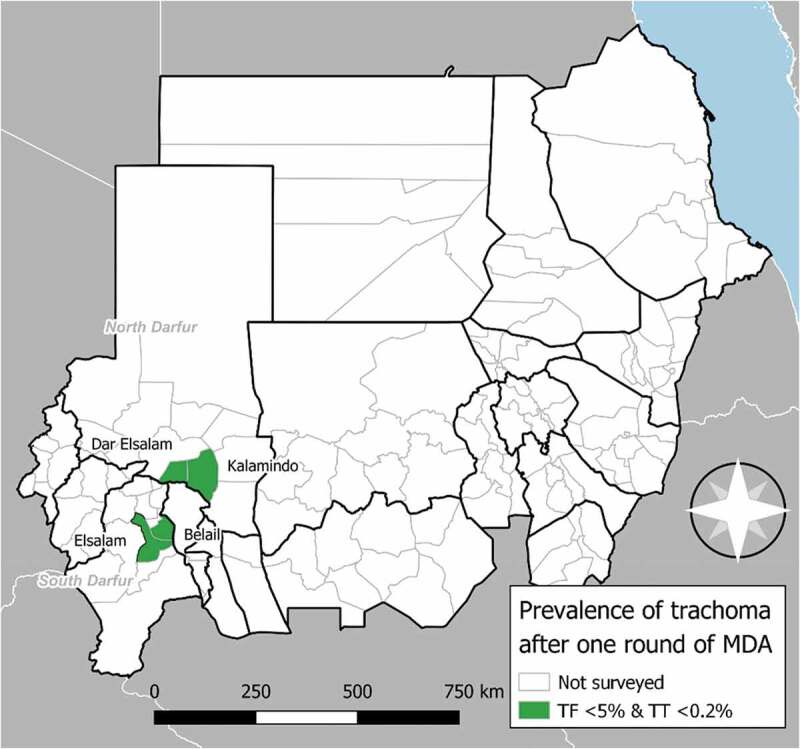
Prevalence of trachomatous inflammation – follicular (TF) in 1–9-year-olds and trachomatous trichiasis (TT) unknown to the health system in ≥15-year-olds in four evaluation units of Sudan. A single round of azithromycin mass drug administration (MDA) took place in these localities in 2017 and the surveys were completed in December 2018–January 2019. The boundaries and names shown and the designations used on this map do not imply the expression of any opinion whatsoever on the part of the authors, or the institutions with which they are affiliated, concerning the legal status of any country, territory, city or area or of its authorities, or concerning the delimitation of its frontiers or boundaries.

In 4,557 ≥15-year-olds examined, 27 cases of TT were identified. Twenty-one (78%) of these cases had TS, and 10 (37%) were affected bilaterally. Twenty-four (89%) of the individuals with TT were ≥50 years in age. Twenty (78%) reported never having previously been offered management for their TT and were therefore classed as “unknown to the health system.” The adjusted prevalence of TT unknown to the health system in ≥15-year-olds was <0.2% in each EU surveyed (range: 0.04–0.19%). EU-level prevalence data are summarised in Table 2 and visualised in Figure 2.

**Table 2.** Prevalence of trachomatous inflammation – follicular and trachomatous trichiasis in four evaluation units (EUs) of Darfur region, Sudan, December 2018–January 2019.

State	Locality	Number of 1–9-year-olds examined	Number of TF cases*	Adjusted prevalence of TF, % (95% CI)	Number of ≥15-year-olds examined	TT^¶^ with scarring*	TT*^¶^	TT^¶^ unknown to the health system	Adjusted prevalence of TT^¶^ unknown to the health system, % (95% CI)
North Darfur	Dar Elsalam	1,253	43	2.7 (1.2–4.8)	1,176	3	3	3	0.08 (0.00–0.17)
	Kalamindo	1,334	47	2.5 (1.4–3.8)	1,100	6	6	4	0.11 (0.00–0.31)
South Darfur	Elsalam	1,461	45	2.4 (1.1–4.2)	1,080	3	4	3	0.04 (0.00–0.09)
	Belail	1,611	62	3.7 (2.1–5.1)	1,201	9	14	10	0.19 (0.03–0.44)
*Total*		*5,659*	*197*	-	*4,557*	*21*	*27*	*20*	-

TF: trachomatous inflammation—follicular; TT: trachomatous trichiasis.

* In one or both eyes.

^¶^In this survey, TT was graded when people were found to have one or more eyelashes touching the eyeball or evidence of recent epilation of in-turned lashes, as per the simplified grading scheme.^2^ Upper- and lower-lid trichiasis were not differentiated.

### Water, sanitation and hygiene access

Water, sanitation and hygiene access was limited in all EUs. Overall, 39% of the households across all four EUs had access to an improved drinking water source within a 30-minute return journey of the house. The availability of improved latrines was particularly low, with 7% of households across all four EUs having an improved latrine (either shared or private). In general, fewer households visited in the post-MDA surveys had access to an improved latrine, to an improved washing water source, and to an improved washing water source within a 30-minute return journey of the house compared to the pre-MDA period (Figure 1). Only 14% of households had a handwashing station within 15 m of their latrine at the time of the visit (Table 3).

**Table 3.** Water, sanitation, and hygiene infrastructure coverage in four evaluation units (EUs) of Darfur region, Sudan, showing both the pre-antibiotic mass drug administration (MDA) data^6^ and post-MDA impact survey data (December 2018–January 2019). Improvement status of latrines and water sources was classified using United Nations Childrens' Fund/World Health Organization Joint Monitoring Programme definitions.^16^ Handwash station data were recorded where there was a station within 15 m of an improved or unimproved latrine but not where there was no latrine.

State	Locality	Clusters (Pre-MDA)	Households (Pre-MDA)	Clusters (Post-MDA)	House-holds (Post-MDA)	Households with improved drinking water source within a 30-minute return journey of the house (%)		Households with an improved latrine (%)		Households with handwashing station within 15 m of the latrine* (%)	
						Pre-MDA	Post-MDA	Pre-MDA	Post-MDA	Pre-MDA	Post-MDA
North Darfur	Dar Elsalam	20	596	25	750	296 (50)	209 (28)	21 (3.5)	52 (7)	408 (68)	63 (8)
	Kalamindo			25	746		148 (20)		87 (12)		87 (12)
South Darfur	Elsalam	20	598	25	749	539 (90)	317 (42)	129 (22)	36 (5)	450 (75)	96 (13)
	Belail			25	749		497 (66)		46 (6)		183 (24)

## Discussion

Excellent progress against trachoma has been made in these four EUs, with TF and TT prevalence in each EU below the respective targets for elimination of trachoma as a public health problem. The four EUs surveyed do not at present qualify for further antibiotic MDA for trachoma elimination purposes. This is a promising result after one round of antibiotic MDA, matching experience elsewhere.^17–19^ These Darfur EUs should be re-surveyed in December 2020–January 2021 following 2 years of F and E interventions but without further MDA, to determine whether the low prevalence of TF measured here has been maintained. The low prevalence of TT in these EUs suggests that active case finding may not currently be necessary. However, the majority of TT cases identified were unknown to the health system, and a system for identifying and managing TT patients is one of the three criteria for validation of elimination,^5^ so these EUs should not be exempted from current nationwide efforts to build surgical capacity for TT.

Darfur residents are known to face significant challenges in accessing water, and improvement of WASH infrastructure coverage is a priority for Sudan’s government and its partners.^20^ The WASH data collected during these surveys highlight the need for the environmental improvement component of the SAFE strategy. Access to WASH recorded in these EUs was generally in line with other contemporary estimates in Sudan. For example, the proportion of households with access to an improved drinking water source within a 30-minute round trip was comparable to recent national-level estimates from rural areas.^16^ The proportion of households with a private, improved latrine was also similar to national estimates.^16^ Moreover, compared to the results from the Global Trachoma Mapping Project baseline surveys in these localities,^6^ a lower proportion of the households surveyed after MDA had access to improved WASH facilities. Darfur has been subject to major armed conflict for a number of decades, and between 2010 and 2017, over 1.6 million people were internally displaced from Darfur due to the destruction of villages and shelters and ongoing violence.^21^ This has likely contributed to the reduction in access to improved WASH facilities, through direct destruction and greater use of limited existing WASH facilities.^22^

Given the association of trachoma with reduced access to water for WASH and the proximity of this population to other populations with high trachoma prevalence, a higher prevalence of disease may have been expected. It is possible that if the observed decline in WASH access is not addressed and MDA treatments are discontinued, recrudescence of trachoma may occur in the future.^23^ The strong association of greater sanitation coverage with lower TF prevalence in children has been demonstrated using data from standardised trachoma surveys undertaken in 13 countries,^24^ and pilot studies in Ethiopia suggest the potential benefit of using soap when face-washing.^25^ However, trachoma prevalence is no doubt influenced by many complex interacting factors, which were not evaluated by this survey methodology. For example, the migration of large numbers of untreated people into areas that have completed their designated rounds of MDA has been shown to increase the risk of reinfection with trachoma.^26^ Considering the political instability of the Darfur region, it is highly likely that further migration events will occur, and thus continued trachoma surveillance of this area will be necessary if the prevalence of TF is to be maintained below the 5% threshold for elimination.

One key limitation of this study is the sample size used to estimate TT prevalence. We examined adults residing in houses selected to assess TF prevalence. Had these surveys been specifically designed to estimate TT prevalence, we would have expanded the number of clusters selected for each EU from 25 to 30 as recommended by the WHO.^27^ We acknowledge that the confidence intervals around each TT prevalence estimate are relatively wide.

Nationwide trachoma control efforts continue in Sudan. In Darfur, neighbouring localities to those studied here had a higher TF prevalence during the most recent mapping exercise^6^; MDA is still ongoing in those districts, and impact surveys are anticipated in 2020–2021. Impact surveys are also warranted in the Blue Nile region, where baseline surveys determined the prevalence of TF in 1–9-year-olds to be ≥10%.^7^ Finally, some localities in the Darfur region, for example, Buram and El Tina, remain unmapped. Baseline surveys are planned in those and other non-surveyed districts to identify whether interventions are required, although ongoing security concerns are likely to make this challenging. While many countries, including Sudan, have now missed the original elimination target of 2020, Sudan has a good chance of eliminating trachoma as a public health problem within the next few years.
